# Clinical Notes on Rickets

**Published:** 1900-03-03

**Authors:** G. Parker

**Affiliations:** Assistant Physician, Bristol General Hospital, Lecturer on Forensic Medicine, Bristol University College.


### Hospital Clinics and Medical Progress.

### CLINICAL NOTES ON RICKETS.

By G. Parker, M.A., M.D.Cantab., Assistant Physician, Bristol General Hospital, Lecturer on Forensic Medicine, Bristol University College.

Probably one-third or more of the entire population have suffered from this disease between the ages of six months and two years. Now, though the direct mortality is small, the final effect on the death-rate of the country is enormous through pulmonary, nervous, and intestinal complications. After recovery, too, a large part of the sufferers remain stunted in growth, perhaps some four inches shorter than they would have been, and the females are subject to future dangers in parturition from contracted pelves. Thus the common error of the student is to regard this disease as an affection of the bones only, in which our chief aim should be to remove their deformities. As a matter of fact this is the least serious and fatal consequence of an attack, for, as Cheadle points out, there is an extraordinary wasting of all the muscular tissues, a weakened and unstable condition of the nervous system often leading to convulsions, spasmodic croup, and tetany, and a catarrhal state of the respiratory and intestinal mucous membranes. During the year or more which an attack lasts ricketty children are in danger from all sorts of illnesses, which would be of little consequence to a normal child, but which frequently carry them off. In large cities it is peculiarly prevalent, but rarer in the open country and in tropical climates. Glisson, in 1650, wrote the first treatise upon it, and from his time it has been frequently called the English malady, though there is no lack of the disease in most populous countries of the Old and New World. James Cooke declared that it was well known before Glisson wrote of it, and discussed its chief symptoms in his "Supplement of the Marrow of Surgery." But even to-day mild cases are frequently overlooked, and the pale pot-bellied child is popularly described as suffering from "consumption of the bowels," or thought to be merely ill-fed. Improper and scanty food no doubt often precedes it, but we are quite in the dark as to the exciting cause, and it is hard to see how the convulsions, catarrhal state, and the thickening and outgrowths of bone are due to inanition. Moreover, it does not appear to be confined to hand-nursed children, or even to those which are brought up in poor households, though much more common in them. The Japanese are said to have a curious immunity, but this may be due to their long seclusion from foreign intercourse, as much as to their habit of suckling their children for five years. They certainly do not use cow's milk at all, though fish oil appears to be given freely to children. Granting that indigestible food or food deficient in fat proteids and phosphates predisposes to rickets, and that bad air, dark rooms, or damp are additional depressing factors, it would seem that none of these of themselves suffice without some exciting cause. The changes in the cartilages and the occasional cirrhotic state of the liver are like the effects of an inflammatory reaction. Much has been made of the fact that young lions, tigers, and monkeys in confinement are very subject to the disease, and quickly recover when milk, fat, and crushed bones are given them freely. Yet some cases of pernicious anæmia in human beings do the same, and we do not imagine that disease to be a result of bad food. The Italians are very little subject to it in their sunny clime, but when they migrate to tenement-houses in New York they seem to suffer worse than other races; yet their food in America is probably richer than in their poverty-stricken country. Apart from the denizens of zoological gardens, it has not been possible to produce rickets in animals by simple starvation, but some of the special changes have been observed after the administration of phosphorus or lactic acid. Hence it has been argued that it is due to lactic acid dyspepsia in young children. But there are, on the other hand, cases where no dyspepsia has been observed, and there are some instances of fœtal or congenital rickets where dyspepsia is out of the question. Hence we can hardly accept the theories of inanition or chronic gastritis. In the osteomalacia of pregnant women, which is thought to be due to the loss of phosphates drained off for the nutrition of the fœtus, the bones are softened, but there the resemblance ends. Hence it cannot be a want of phosphates which is the cause, and phosphates too are plentiful in the meal foods on which some ricketty children have been fed. Much can be said in favour of some irritating toxin as the agent, though its nature is at present quite unknown. Nor can we say that the disease is directly infectious, though affecting special communities. In many respects we are reminded of the effects of the tuberculous toxin, though there is said to be often a complete absence of pyrexia. Thus the sweats, the wasting, the anæmia are the same in both cases, as well as the results of good feeding, light, and fresh air. Very occasionally bone changes, too, are seen in tuberculous patients not unlike those seen in rickets. Parret broached the theory that it is a form of syphilis, but though some of the changes in the skull are hard to distinguish, and one disease may concur with and modify the effects of the other, it is abundantly clear that most ricketty children are free from the taint of syphilis, and this latter disease never affects several of the animals, such as young lions, which are very subject to rickets. Perhaps the only disease of adult life, where bony outgrowths are found at all comparable to those of rickets, is rheumatoid arthritis, and here there is strong reason to believe in the irritative action of a toxin produced in the tissues by a bacillus which is absorbed through the intestines or uterus. However, there is no softening of the bone. In rickets, indeed, it has been said, the distinguishing feature above all others is the excessive promise of bone formation, and the defective performance. There are enlargements at the junction of the ribs and cartilages, at the ends of the long bones, such as near the wrists and ankles, and there are thickenings in the skull, giving it a quadrangular shape. The bones themselves soften and bend under the weight of the body, the tibiæ, humeri, radii, ulnæ and the clavicles bend forwards, and the tibiæ outwards also. The ribs give way from the strain of breathing and the tension of the diaphragm, leaving a huge depression on the chest in the line where the nodes can be felt. The ligaments also yield, causing flat foot, spinal curvature, and ill-shaped joints. The teeth are late and irregular in appearing and decay early, the head is often covered with profuse sweats. The child is restless by night and drowsy and fretful by day. He fears to move or to be handled because his bones are tender. Frequent attacks of dyspepsia and diarrhœa, with a huge swollen abdomen, are common, and may be accompanied with an enlarged fibrotic liver and spleen in some few cases. The failure of the ribs and the bronchial troubles lead to collapse of the lung, and are rendered more dangerous by the frequency of spasmodic croup. Hence, the child is brought to a doctor for all sorts of complaints except the real malady, which lies at the root of his various symptoms. He is thought to have infantile paralysis because of his weak and tender limbs; or disease of the spine, because of its curvature from weakness without any angular deformity; or a deformed breast bone from the falling in of his ribs. His wasting, diarrhœa, convulsions, or croup may in other cases be the cause of his appearance, and most often of all whatever symptom occurs is put down as a result of teething or vaccination. As to treatment, if we can improve the nutrition the disease rapidly disappears in most cases, the deformities tend to vanish, and all the complications cease to be important. Probably we shall have to deal at first with intestinal fermentation and catarrh. Nothing in my experience is so effective for this as the liquor hydrargyri or castor oil, to be followed by bismuth. The latter should be given, according to Cheadle, in full doses of 10 grains each for an infant of six months if diarrhœa continues. At the same time the diet must be regulated, and this is the chief matter, together with fresh air and cleanliness. Up to six or eight months, for hand-fed children, cows' milk diluted with barley water, and, if possible, a little cream added, should be the sole food. Even here a teaspoonful of raw meat juice, or red gravy from a joint, may be given daily, or 30 drops of cod-liver oil twice a day. Similar aids may be administered to a breast-fed child. Above this age malted foods or fine oatmeal must be given in addition to the milk, but the cream or oil and the meat-juice should be continued. Gradual additions are then made to the dietary as time goes on, and if there is any indigestion it must be remedied at once. The evacuations should be watched for undigested curd, or green-fermenting stools, and in the severest cases predigested food may be necessary at first for a few days. As to medicines, we must beware of irritating the stomach, which iodide of iron, though otherwise useful, is almost sure to do. The phosphate so commonly employed is less guilty in this respect, but must be watched for the same reason. Many children, however, mend steadily under it. Guaiacol carbonate or creasote in milk seem worth trying in obstinate cases, or they may be added to the oil. In short, we must, as Cheadle says, see that there is no digestive inability to absorb food; and, secondly, that the food given contains plenty of animal fats, proteid, and phosphates. Complications, such as bronchitis and convulsions, must at the same time be energetically treated, and deformities prevented as much as possible by keeping the child on its back when feasible. Severe forms will, of course, need splints and even surgical treatment.

